# Supplementary material for: Structures of folding intermediates on BAM show diverse substrates fold by a conserved mechanism
Source: Proc Natl Acad Sci U S A. 2026 Apr 2;123(14):e2534936123. doi: 10.1073/pnas.2534936123 (PMC13048289; doi:10.1073/pnas.2534936123)
Supplement: Supplementary file 1 [file pnas.2534936123.sapp.pdf]

PNAS

Supporting Information for

Structures of folding intermediates on BAM show diverse substrates fold by a conserved mechanism

Benjamin D. Thomson[1], Melissa D. Marquez[1], Shaun Rawson[2], Thiago M. A. dos Santos[1]†, Stephen C. Harrison[2,3], and Daniel Kahne[1]

[1]Department of Chemistry and Chemical Biology, Harvard University, Cambridge, MA, USA.

[2]Department of Biological Chemistry and Molecular Pharmacology, Harvard Medical School, Boston, MA, USA.

[3]Howard Hughes Medical Institute, Boston, MA, USA.

†Present address: Department of Chemistry and Chemical Biology, Northeastern University, Boston, MA, USA.

Corresponding authors: Daniel Kahne and Stephen C. Harrison

Email: kahne@chemistry.harvard.edu or harrison@crystal.harvard.edu

This PDF file includes:

Supporting text
Figures S1 to S10
Tables S1 to S4
SI References

Figures

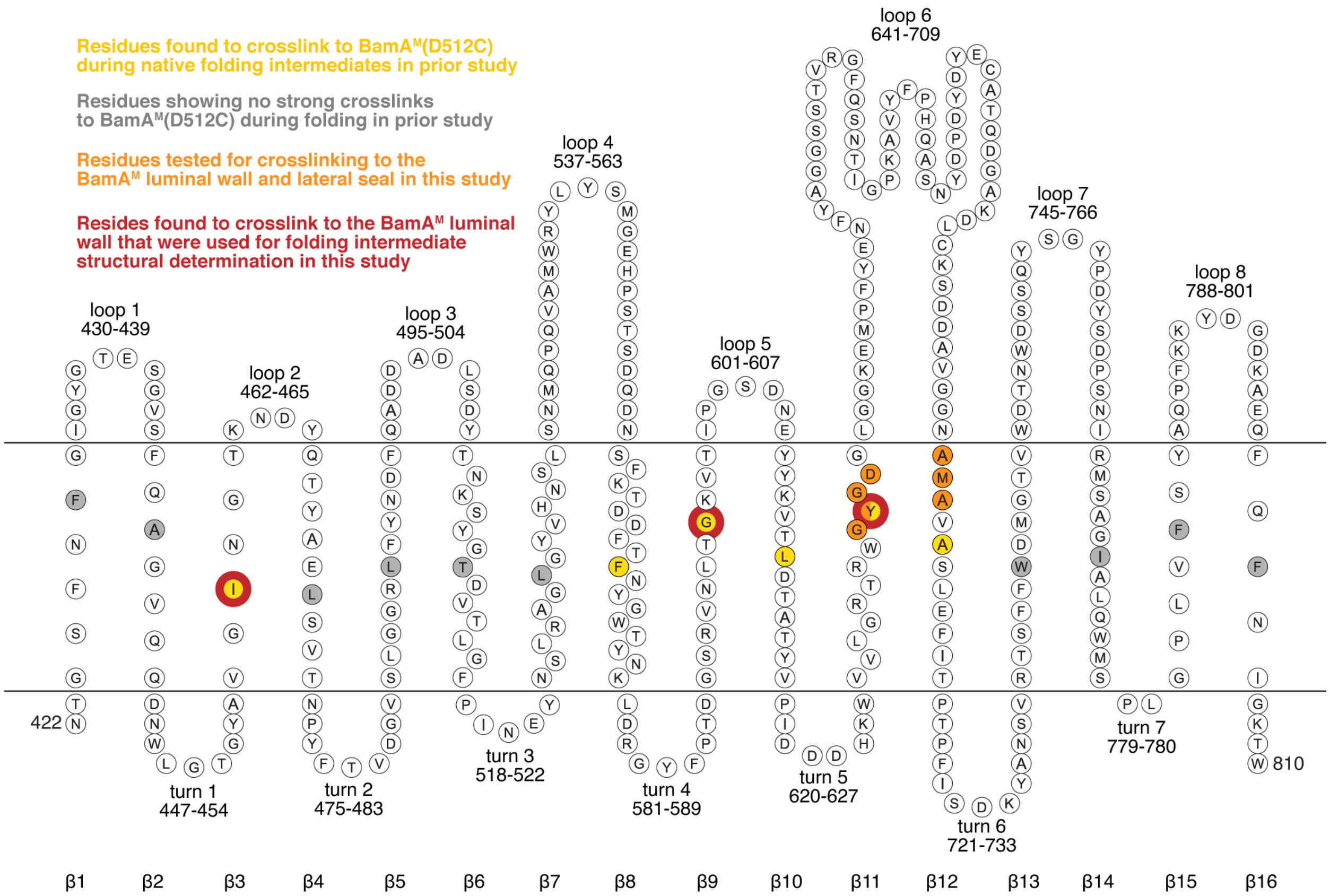

Fig. S1: Topological depiction of the BamA β-barrel domain. The extracellular loops, periplasmic turns, and transmembrane β-strands are labeled. Residues within BamA^S β-strands that, upon substitution with cysteine, were previously found to crosslink to BamA^M(D512C) during native folding intermediates are highlighted in yellow (1). Tested residues within β-strands that were found to show no such strong crosslinking are shown in gray. Residues that were substituted for cysteines and used in crosslinking experiments to the BamA^M luminal wall to the BamA^M C-terminal region at the lateral seal are highlighted in orange. Residues that were substituted for cysteines to trap folding BamA^S within native folding intermediates for structural characterization are outlined with red circles. These substitutions were used to purify, from left to right, the Late Intermediate (BAM-BamA^S(I458C)), Middle Intermediate (BAM-BamA^S(G597C)), and Early Intermediate (BAM-BamA^S(Y637C)) complexes.

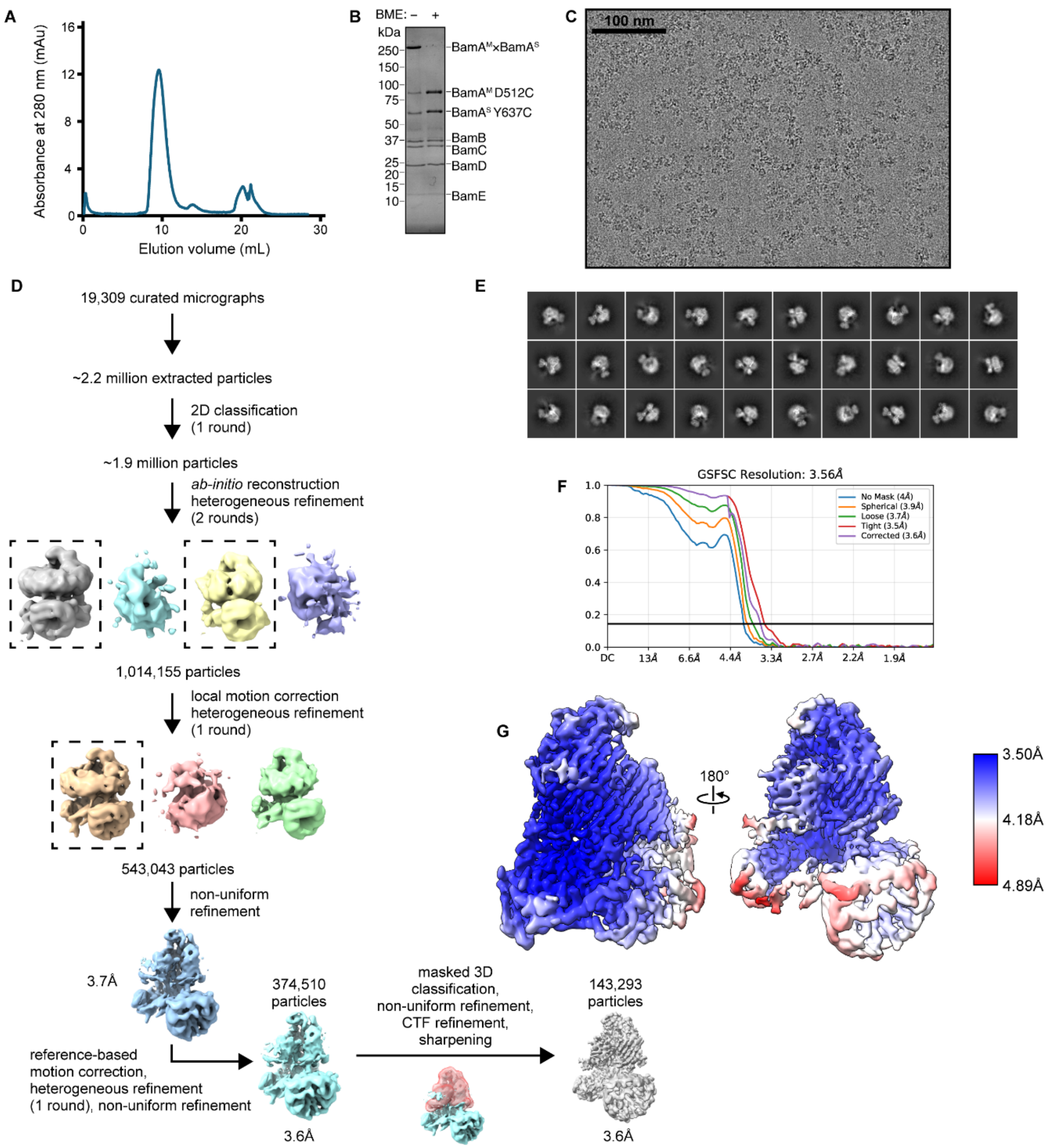

Fig. S2. Cryo-EM data processing and analysis for the Early Intermediate complex. (**A**) Representative size-exclusion chromatogram of the Early Intermediate complex. The major peak fractions were collected and pooled before concentration and freezing for cryo-EM grid preparation. (**B**) Coomassie-stained SDS-PAGE gel of purified Early Intermediate complex. The high-molecular-weight band present within the sample reduces to BamA^{M} and BamA^{S} upon addition of β-mercaptoethanol (BME). (**C**) Representative cryo-EM micrograph of the Early Intermediate complex embedded in vitreous ice. (**D**) Scheme of cryo-EM data processing for the Early Intermediate complex. Images of cryo-EM densities were created using ChimeraX. Focused 3D classification used a mask that surrounded the hybrid barrel region of the density. (**E**) Representative 2D class averages of extracted Early Intermediate cryo-EM particles. (**F**), Gold-standard Fourier shell correlation (FSC) curves calculated with different masks within cryoSPARC.

The resolution was determined at FSC = 0.143 (horizontal line). The final corrected mask gave a resolution of 3.6Å. (**G**), Final map of the Early Intermediate non-uniform refined density. The density has been colored by local resolution calculated using RELION. The map used for model building and validation was sharpened with a uniform B-factor of -100. Maps displayed in the main text figures were postprocessed with DeepEMhancer.

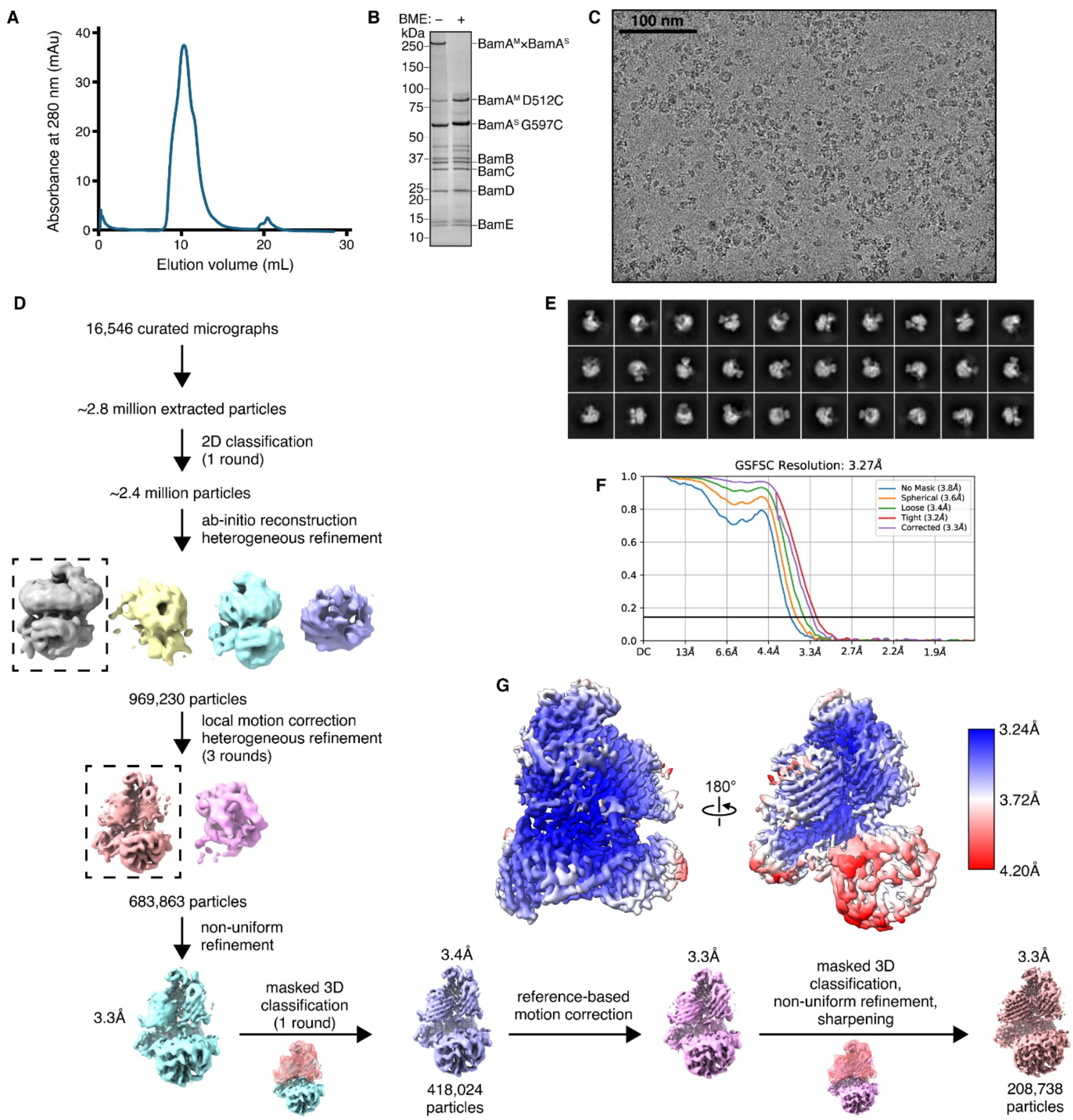

Fig. S3: Cryo-EM data processing and analysis for the Middle Intermediate complex. (A) Representative size-exclusion chromatogram of the Middle Intermediate complex. The major peak fractions were collected and pooled before concentration and freezing for cryo-EM grid preparation. (B) Coomassie-stained SDS-PAGE gel of purified Middle Intermediate complex. The high-molecular-weight band present within the sample reduces to BamA^M and BamA^S upon addition of β-mercaptoethanol (BME). (C) Representative cryo-EM micrograph of the Middle Intermediate complex embedded in vitreous ice. (D) Scheme of cryo-EM data processing for the Middle Intermediate complex. Images of cryo-EM densities were created using ChimeraX. Focused 3D classification used a mask that surrounded the hybrid barrel region of the density. (E) Representative 2D class averages of extracted Middle Intermediate cryo-EM particles. (F) Gold-standard Fourier shell correlation (FSC) curves calculated with different masks within cryoSPARC. The resolution was determined at FSC = 0.143 (horizontal line). The final corrected mask gave a resolution of 3.3Å. (G) Final map of the Middle Intermediate non-uniform refined density. The density has been colored by local resolution calculated using RELION. The map used for model

building and validation was sharpened with a uniform B-factor of -115. Maps displayed in the main text figures were postprocessed with DeepEMhancer.

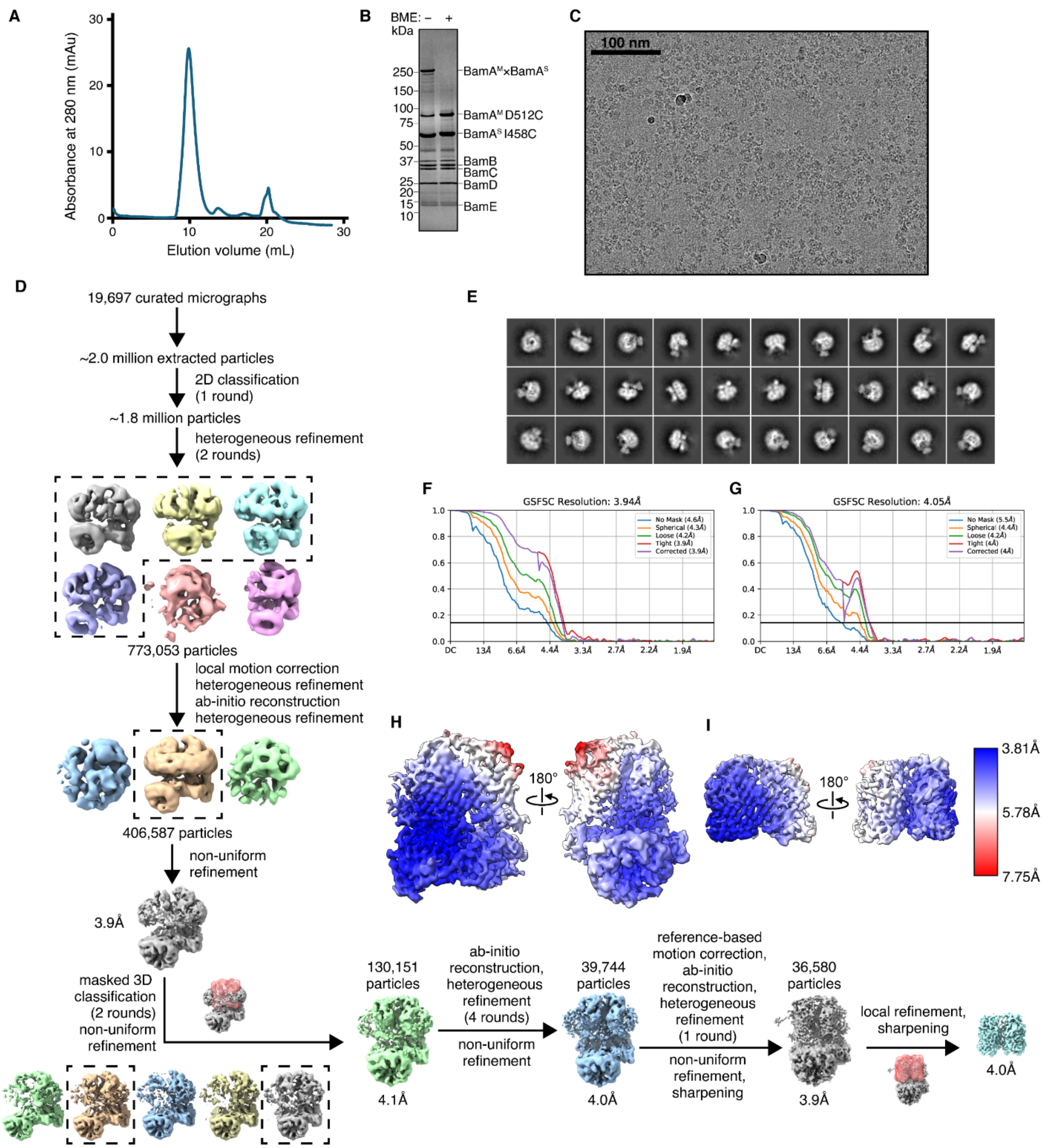

Fig. S4: Cryo-EM data processing and analysis for the Late Intermediate complex. (**A**) Representative size-exclusion chromatogram of the Late Intermediate complex. The major peak fractions were collected and pooled before concentration and freezing for cryo-EM grid preparation. (**B**) Coomassie-stained SDS-PAGE gel of purified Late Intermediate complex. The high-molecular-weight band present within the sample reduces to BamA^M and BamA^S upon addition of β-mercaptoethanol (BME). (**C**) Representative cryo-EM micrograph of the Late Intermediate complex embedded in vitreous ice. (**D**) Scheme of cryo-EM data processing for the Late Intermediate complex. Images of cryo-EM densities were created using ChimeraX. Focused 3D classification and local refinement masks surrounded the hybrid barrel region of the density. (**E**) Representative 2D class averages of extracted Late Intermediate cryo-EM particles. (**F, G**) Gold-standard Fourier shell correlation (FSC) curves calculated with different masks within cryoSPARC from the global

non-uniform refined (F) and locally refined (G) densities of the Late Intermediate complex. The resolution was determined at FSC = 0.143 (horizontal line). The final corrected mask gave a resolution of 3.9Å for the global non-uniform refined density, and a resolution of 4.1Å for the locally refined density. (**H, I**) Final maps of the Late Intermediate non-uniform refined density (H) and locally refined density (I). The density has been colored by local resolution calculated using RELION. Final maps used for model building were individually sharpened with a uniform B-factor of -141 and combined into a composite map using PHENIX. Maps displayed in the main text figures were postprocessed with DeepEMhancer and then combined using PHENIX.

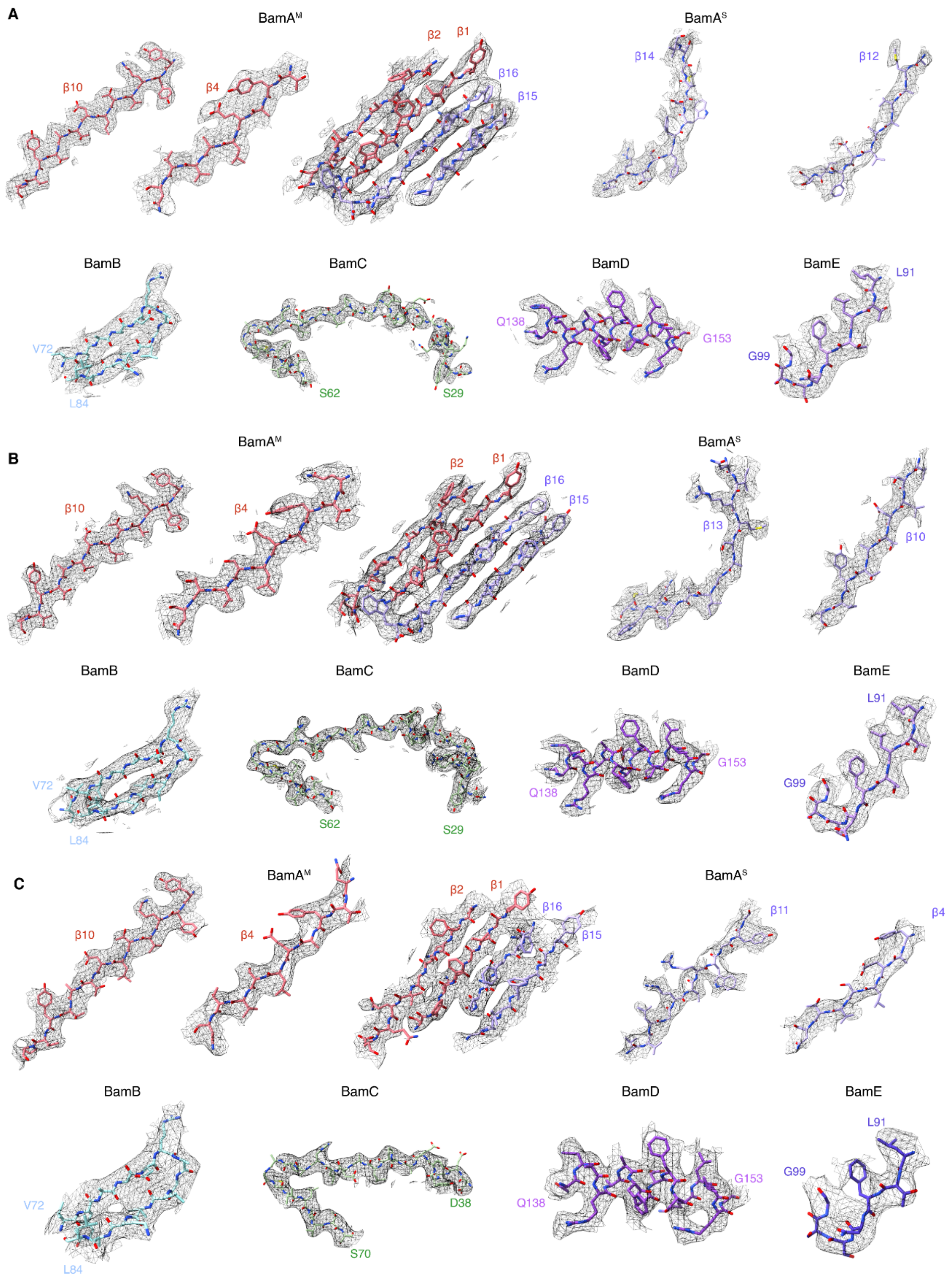

Fig. S5: Fit of the atomic models into the BamA^{S} intermediate cryo-EM maps. (**A-C**) Atomic models and their fit into the sharpened cryo-EM maps for selected parts of each chain in the Early Intermediate (A), the Middle Intermediate (B), and the Late Intermediate (C) structures. Images were generated in ChimeraX using a carve radius of 2.5Å.

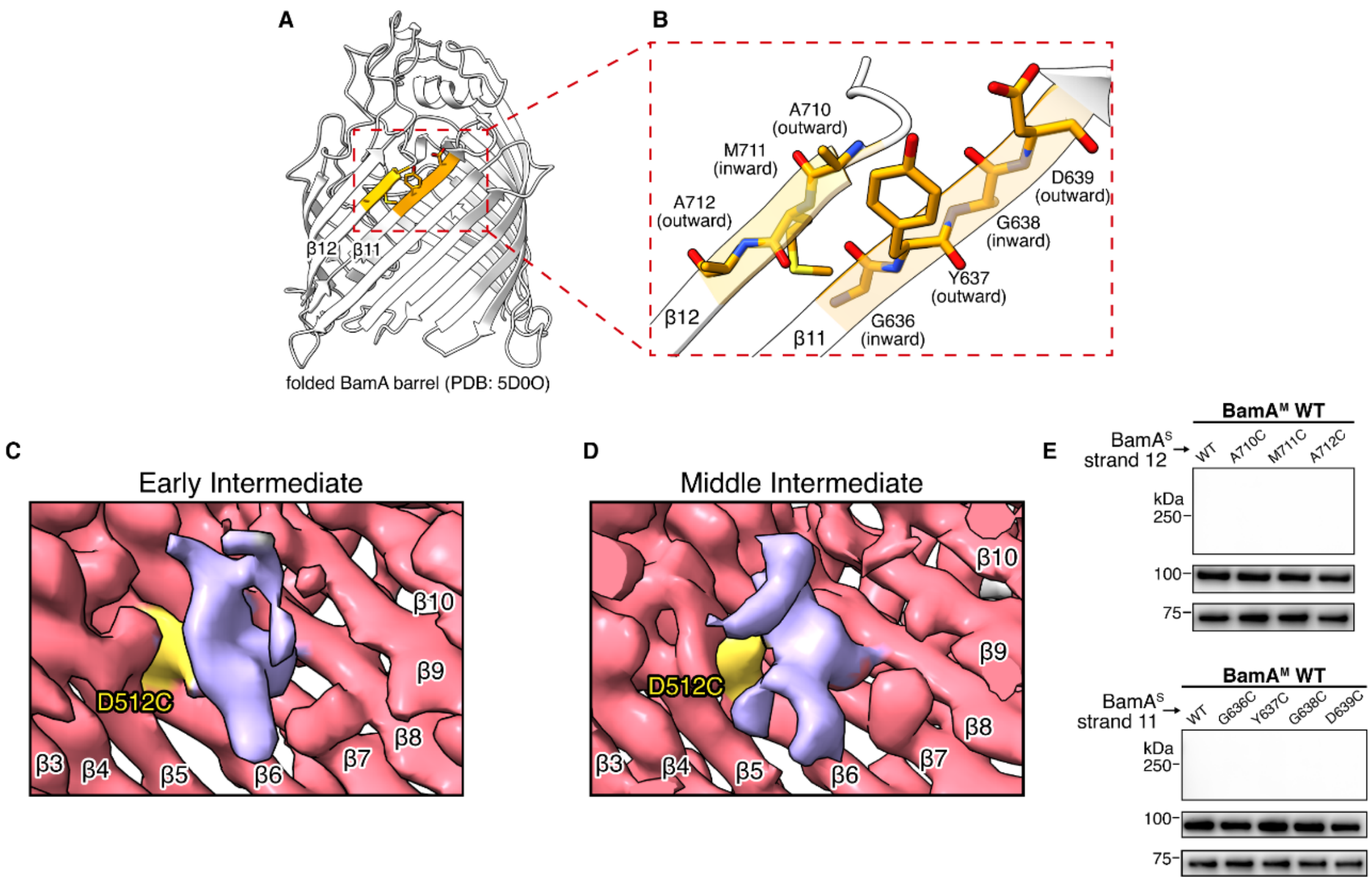

Fig. S6: BamA^{S} density within the BamA^{M} barrel. (**A**), Locations of β-strands 11 and 12 within the closed, folded BamA barrel structure. The residues within β-strands 11 and 12 that were substituted for cysteines and used for disulfide crosslinking experiments to the BamA^{M} luminal wall and to the BamA^{M} C-terminal region at the lateral seal are highlighted in orange. (**B**), Region of the folded BamA barrel that contains the residues tested for luminal wall and lateral seal disulfide crosslinking. When organized into the native sheet, A710 and A712 within β-strand 12 have outward-facing side chains pointed toward the membrane while M711 has an inward-facing side chain pointed toward the barrel lumen. Similarly, when organized into the native sheet, Y637 and D639 have outward-facing side chains while G636 and G638 would have inward-facing side chains upon substitution with cysteine. (**C**, **D**) Inside-the-barrel view of the BamA^{M} luminal wall for the Early Intermediate (C) and Middle Intermediate (D) cryo-EM densities. BamA^{M} strands are labeled. Both maps contain density within the BamA^{M} barrel at the density at the point of crosslinking between the machine and substrate: BamA^{M}(D512C) (yellow). We interpret this density as corresponding to the disordered region of the substrate containing the crosslinking substrate cysteine in each complex (β-strand 11 for the Early Intermediate, and β-strand 9 for the Middle Intermediate). (**E**) Expression of BamA^{S} cysteine substitutions in β-strand 12 (upper) and β-strand 11 (lower) alongside wild-type BamA^{M} containing no cysteine substitution results in no disulfide crosslinking bands. Disulfide crosslinks between BamA^{M}(D512C) or BamA^{M}(I806C) and BamA^{S} cysteine-containing variants are therefore dependent upon the engineered BamA^{M} cysteine. Data are laid out as described in Fig. 2D.

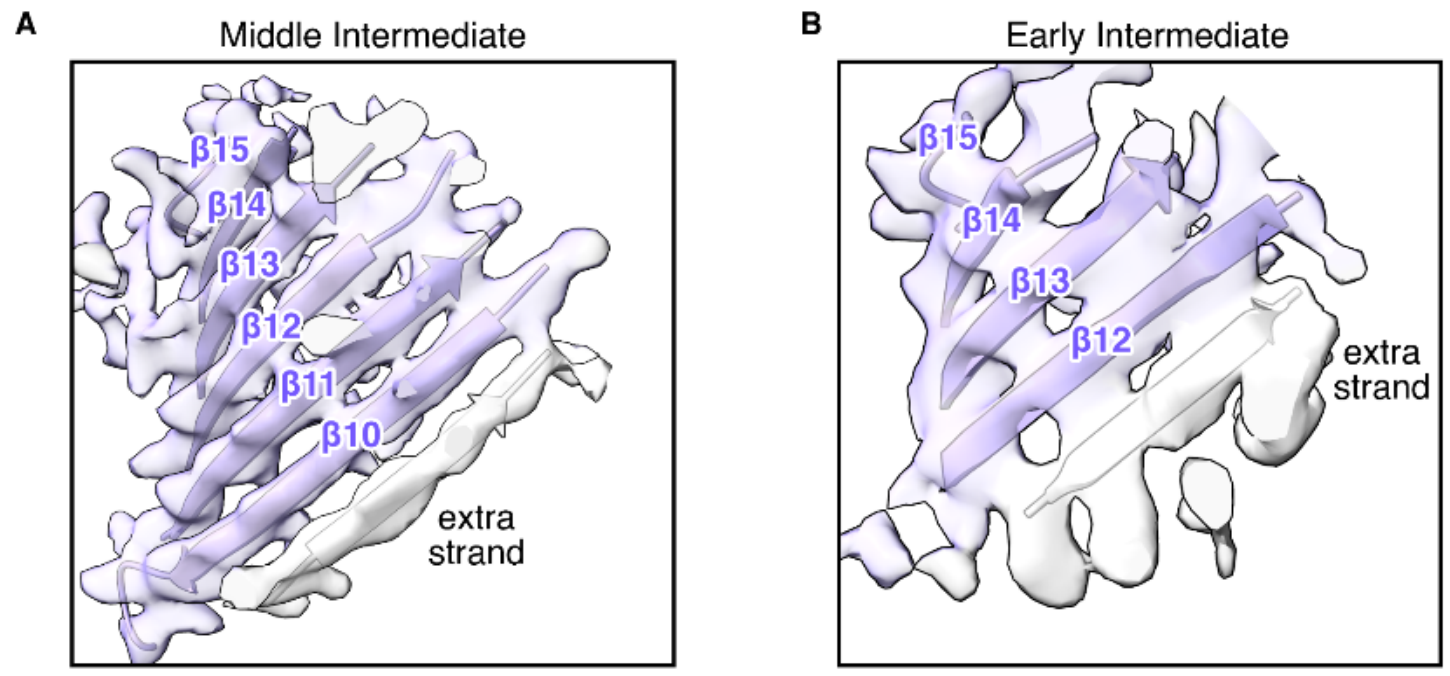

Fig. S7: Substrate density in the Early and Middle Intermediate structures. (**A**, **B**) Detail view of the $BamA^S$ substrate density at the interface of the substrate edge and the $BamA^M$ lumen for the Middle Intermediate (A) and Early Intermediate (B). An "extra strand" of density is visible for each intermediate, but not featured enough to clearly identify (light grey). A polyalanine β-strand sequence was built into this density in the Early and Middle Intermediate models.

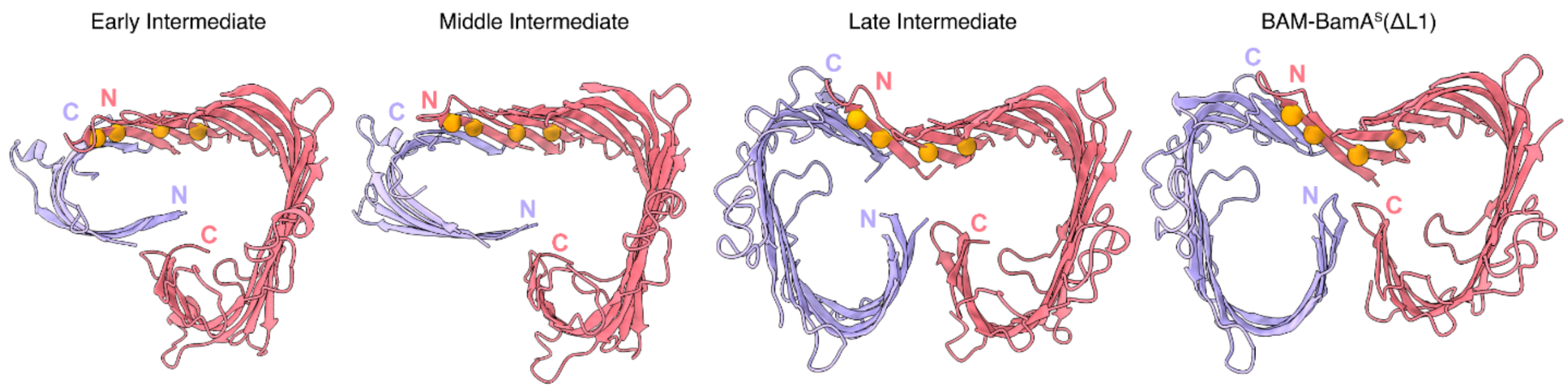

Fig. S8: The region of BamA^{M} that flexes outward contains several inward-oriented glycines. Top-down depictions of the hybrid barrel domains of, from left to right, the Early Intermediate, Middle Intermediate, Late Intermediate, and BAM-BamA^{S}(ΔL1). Four inward-oriented glycine residues within BamA^{M} β-strands 1-3, the machine's N-terminal β-strands that progressively flex outward during folding, are indicated (orange spheres). Several extracellular loops have been hidden for clarity.

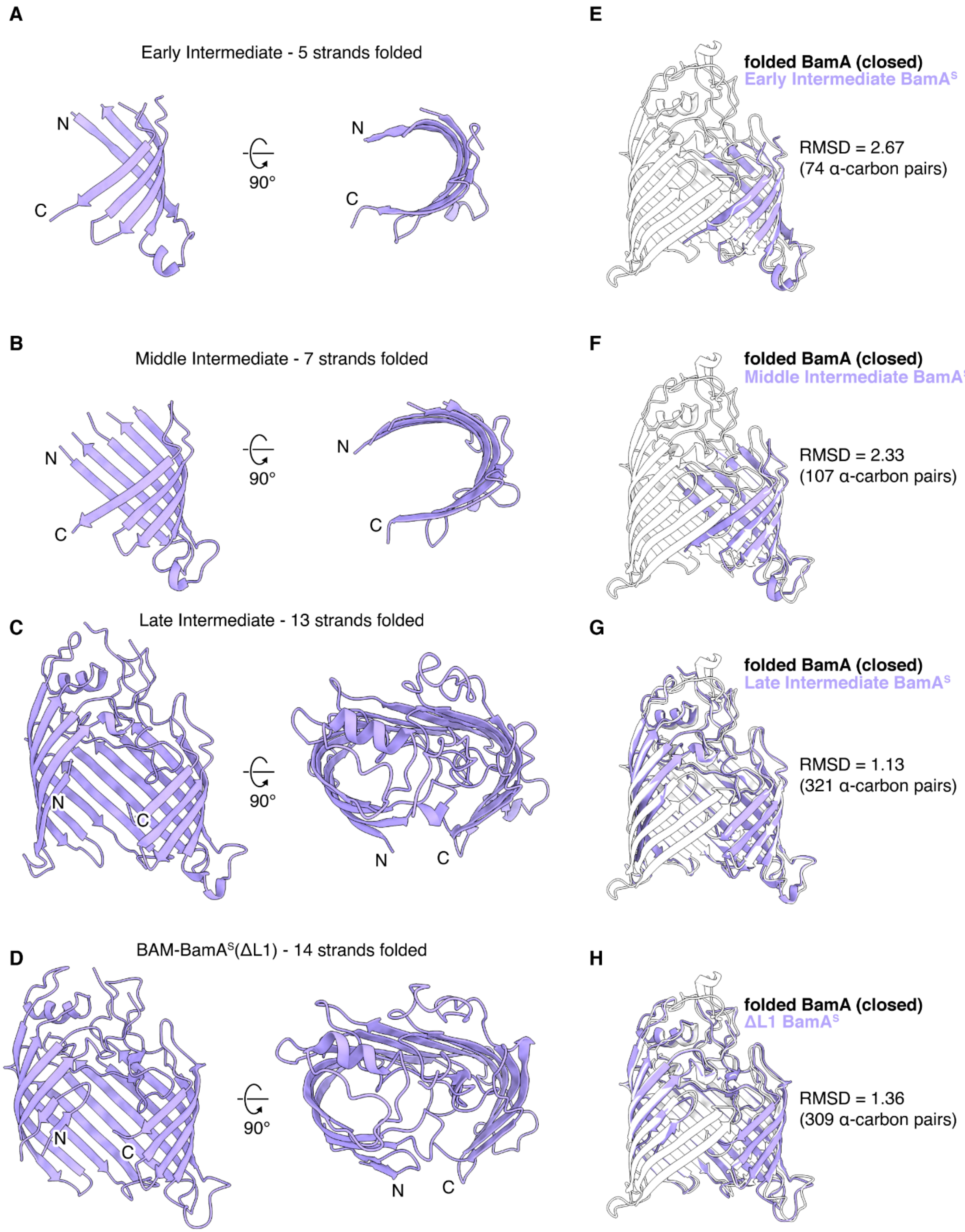

Fig. S9: Folded portions of the BamA^S substrate barrel in progressive folding intermediates. (**A-D**), Side view (left) and top-down view (right) of the BamA^S substrate barrel within the Early Intermediate (A), Middle Intermediate (B), Late Intermediate (C) and BAM-BamA^S(ΔL1) (D)

structures. As strands are added, the substrate's open N-terminal edge approaches the C-terminal strand. (**E**-**H**) Overlays of the fully folded closed BamA barrel (white, PDB: 5D0O) and the $BamA^S$ substrate (lavender) for the Early Intermediate (E), Middle Intermediate (F), Late Intermediate (G) and BAM-$BamA^S$(ΔL1) (H). Overall, the substrate's folded strands closely resemble the corresponding portion of the folded, closed $BamA^S$ barrel throughout folding.

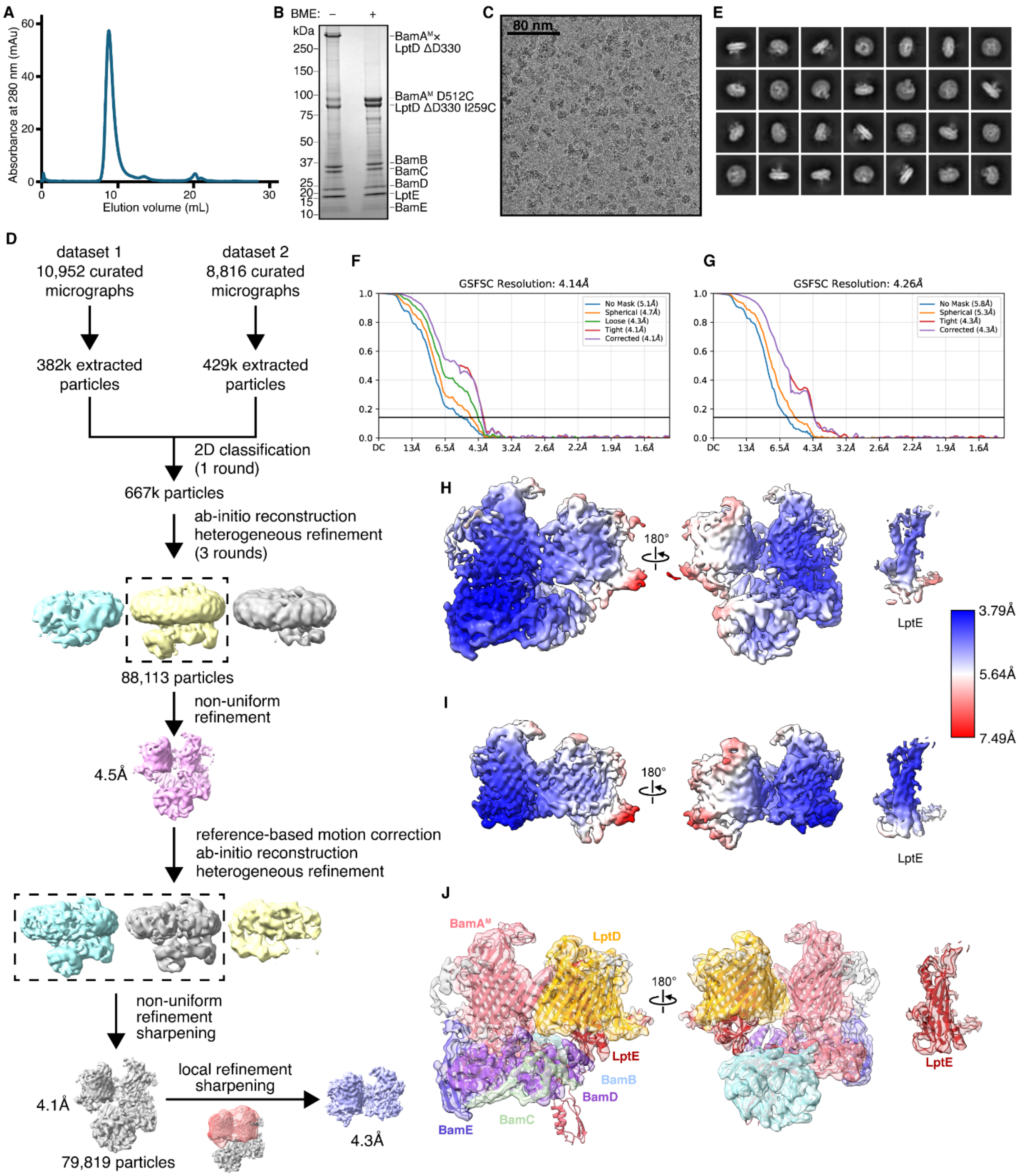

Fig. S10: Cryo-EM data processing and analysis for the Late LptD Intermediate complex. (**A**) Representative size-exclusion chromatogram of the Late LptD Intermediate complex. The major peak fractions were collected and pooled before concentration and freezing for cryo-EM grid preparation. (**B**) Coomassie-stained SDS-PAGE gel of purified Late LptD Intermediate complex. The high-molecular-weight band present within the sample reduces to BamA^M and LptD upon addition of β-mercaptoethanol (BME). (**C**) Representative cryo-EM micrograph of the Late LptD Intermediate complex embedded in vitreous ice. (**D**) Scheme of cryo-EM data processing for the Late LptD Intermediate complex. Images of cryo-EM densities were created using ChimeraX. The local refinement mask surrounded the hybrid barrel region of the density, including the LptE plug.

(**E**) Representative 2D class averages of extracted Late Intermediate cryo-EM particles. (**F, G**) Gold-standard Fourier shell correlation (FSC) curves calculated with different masks within cryoSPARC from the global non-uniform refined (F) and locally refined (G) densities of the Late Intermediate complex. The resolution was determined at FSC = 0.143 (horizontal line). The final corrected mask gave a resolution of 4.1Å for the global non-uniform refined density, and a resolution of 4.3Å for the locally refined density. (**H, I**) Final maps of the Late LptD Intermediate non-uniform refined density (H) and locally refined density (I). The density has been colored by local resolution calculated within RELION. Final maps used for model building were individually sharpened with a uniform B-factor of -112 and combined into a composite map using PHENIX. Density for the LptE plug produced using a carve radius of 2.5Å around LptE is shown (right). (**J**) Fit of atomic model into the Late LptD Intermediate cryo-EM map. Models and densities corresponding to $BamA^{M}$, BamB, BamC, BamD, BamE, LptD, and LptE are colored in salmon, light blue, light green, purple, dark blue, orange, and red respectively. The composite map of local refined and global refined density is shown. Density for the LptE plug produced using a carve radius of 2.5Å around LptE is shown (right).

Tables

Table S1: Cryo-EM data collection, refinement, and validation statistics

	Early Intermediate (PDB 9MGE) (EMD-48253)	Middle Intermediate (PDB 9MGF) (EMD-48254)	Late Intermediate (PDB 9MGG) (EMD-48255)	Late LptD Intermediate (PDB 9P1U) (EMD-71132)
**Data collection and processing**				
Microscope	Krios	Krios	Krios	Krios
Magnification	105,000	105,000	105,000	165,000
Voltage (kV)	300	300	300	300
Electron exposure (e-/Å^2)	51.1	53	49.6	49.5/52.2
Defocus range (μm)	1.2-2.6	1.0-2.4	1.0-2.4	0.6-2.0
Pixel size (Å)	0.83	0.83	0.83	0.74
Symmetry imposed	C1	C1	C1	C1
Initial particle images (no.)	2,252,778	2,884,911	1,999,604	811,231
Final particle images (no.)	143,293	208,738	39,744	79,819
Global map resolution (Å)	3.6	3.3	3.9	4.1
FSC threshold	0.143	0.143	0.143	0.143
**Refinement**				
Initial model source	5D0O, 5LJO	6V05, 5LJO	5D0O, 5LJO	5LJO, Alphafold
Model resolution (Å)	4.0	3.5	4.2	4.3
FSC threshold	0.5	0.5	0.5	0.5
Map sharpening B-factor (Å^2)	-100	-115	-141	-112
Model composition				
Non-H atoms	11740	12658	13296	15852
Protein residues	1581	1625	1774	2080
Ligands	0	0	0	0
Model vs. data CC	0.75	0.76	0.76	0.76
R.m.s. deviations				
Bond lengths (Å)	0.005	0.003	0.004	0.004
Bond angles (°)	0.933	0.743	0.839	0.722
Validation				
Molprobity score	1.91	1.54	2.02	1.84
Clashscore	7.13	4.35	10.13	10.41
Poor rotamers (%)	1.10	0.37	1.03	0.25
Ramachandran plot				
Favored (%)	92.16	95.25	92.11	95.61
Allowed (%)	7.84	4.62	7.83	4.20
Disfavored (%)	0.00	0.12	0.06	0.20

Table S2. Plasmids used in this study.

Plasmid	Description	Source
pBDT513	pTrc99a/8×His–BamA(D512C)–BamB–BamC–BamD–BamE	This study
pBDT422	pBAD33/2×Strep–BamA(Δ172-421/Y637C)	This study
pBDT382	pTrc99a/BamA(D512C)–BamB–BamC–BamD–BamE–8×His	This study
pBDT393	pBAD33/2×Strep-BamA(Δ172-421/G597C)	This study
pBDT517	pBAD33/2×Strep-BamA(Δ172-421/I458C)	This study
pBDT505	pZS21/6×His–BamA	This study
pBDT374	pZS21/6×His–BamA(D512C)	(*1*)
pBDT507	pZS21/6×His–BamA(I806C)	This study
pBDT510	pTrc99a/3×FLAG-BamA(Δ172-421/A710C)	This study
pBDT523	pTrc99a/3×FLAG-BamA(Δ172-421/M711C)	This study
pBDT511	pTrc99a/3×FLAG-BamA(Δ172-421/A712C)	This study
pBDT500	pTrc99a/3×FLAG-BamA(Δ172-421/G636C)	This study
pBDT501	pTrc99a/3×FLAG-BamA(Δ172-421/Y637C)	(*1*)
pBDT502	pTrc99a/3×FLAG-BamA(Δ172-421/G638C)	This study
pBDT503	pTrc99a/3×FLAG-BamA(Δ172-421/D639C)	This study
pBDT553	pTrc99a/3×FLAG-BamA(Δ172-421/F572C)	(*1*)
pBDT378	pTrc99a/3×FLAG-BamA(Δ172-421/G597C)	This study
pBDT554	pTrc99a/3×FLAG-BamA(Δ172-421/L613C)	(*1*)
pBDT572	pTrc99a/3×FLAG-BamA(Δ172-421/Δ601-607/F572C)	This study
pBDT573	pTrc99a/3×FLAG-BamA(Δ172-421/Δ601-607/G597C)	This study
pBDT574	pTrc99a/3×FLAG-BamA(Δ172-421/Δ601-607/L613C)	This study
pBDT586	pTrc99a/3×FLAG-BamA(Δ172-421/Δ430-439/F572C)	This study
pBDT587	pTrc99a/3×FLAG-BamA(Δ172-421/Δ430-439/G597C)	This study
pBDT588	pTrc99a/3×FLAG-BamA(Δ172-421/Δ430-439/L613C)	This study
pBDT514	pTrc99a/3×FLAG-BamA(Δ172-421/I458C)	(*1*)
pBDT534	pTrc99a/3×FLAG-BamA(Δ172-421/Δ447-454/I458C)	This study
pBDT536	pTrc99a/3×FLAG-BamA(Δ172-421/Δ430-439/I458C)	This study
pBDT538	pTrc99a/3×FLAG-BamA(Δ172-421/Δ537-563/I458C)	This study
pBDT584	pTrc99a/3×FLAG-BamA(Δ172-421/Δ495-504/I458C)	This study
pTS1293	pCDFDuet/LptE/2×Strep–LptD(ΔD330/I259C)	This study

Table S3: Strains used in this study

Strain	Genotype	Source
NovaBlue	*end*A1 *hsd*R17 (r_K^-, m_K^+) *sup*E44 *thi*-1 *rec*A1 *gyr*A96 *rel*A1 *lac* F′[*pro*A^+ B^+ *lac*I^q ZΔM15::Tn*10*]	Novagen
BL21 (DE3)	*fhuA2 [lon] ompT gal (λ DE3) [dcm] ΔhsdS*	Novagen
MC4100	F^- *araD139 Δ(argF-lac)U169 rpsL150 relA1 flbB5301 deoC1 ptsF25 rbsR thi*	(*2*)

Table S4: Oligonucleotides used in this study

Description	Sequence (5' to 3')
BamA β1 Gibson F	cttcaactttggtattggttacgg
BamA β1 Gibson R	ccgtaaccaataccaaagttgaag
BamA β16 Gibson F	catcggtaaaacctggtaagtgg
BamA β16 Gibson R	ccacttaccaggttttaccgatg
BamA(G597C) F	caacctgacctgcaaagtgacc
BamA(G597C) R	ggtcactttgcaggtcaggttg
BamA(I806C) F	gttccagtttaactgcggtaaaacctg
BamA(I806C) R	tgttctgccttgtctccatcgtac
BamA(A710C) F	gcggtaactgcatggcggttg
BamA(A710C) R	ctacagcatcatccgatttacacag
BamA(M711C) F	gtaacgcctgcgcggttgcc
BamA(M711C) R	cgcctacagcatcatccgatttac
BamA(A712C) F	gtaacgccatgtgcgttgccag
BamA(A712C) R	cgcctacagcatcatccgatttac
BamA(G636C) F	ccgctggtgctatggtgatgg
BamA(G636C) R	gtacgccccagaacaaccc
BamA(G638C) F	gggttattgcgatggtttaggcgg
BamA(G638C) R	cagcgggtacgccccag
BamA(D639C) F	ggttatggttgcggtttaggcggc
BamA(D639C) R	ccagcgggtacgcccc
BamA(Δ601-607) F	tactacaaagtgacgttagacacgg
BamA(Δ601-607) R	ggtcactttaccggtcaggttg
BamA(Δ430-439) F	ttccaggctggtgtgcagc
BamA(Δ430-439) R	accaaagttgaagctaccggtg
BamA(Δ447-454) F	gctgttggtatcaacgggacc
BamA(Δ447-454) R	ctgctgcacaccagcctg
BamA(Δ537-563) F	agcttcaaaacggacgacttcacg
BamA(Δ537-563) R	cagggagttatgtacataacccag
BamA(Δ495-504) F	cttctataatgacttccagaccaacaagagttatgg
BamA(Δ495-504) R	ccataactcttgttggtctggaagtcattatagaag

SI References

1. T. M. A. dos Santos, et al., Native β-barrel substrates pass through two shared intermediates during folding on the BAM complex. Proc. Natl. Acad. Sci. 121, e2409672121 (2024).
2. M. J. Casadaban, Transposition and fusion of the lac genes to selected promoters in Escherichia coli using bacteriophage lambda and Mu. J. Mol. Biol. 104, 541–555 (1976).
